# Challenges to the ΛCDM cosmology

**DOI:** 10.1098/rsta.2024.0022

**Published:** 2025-02-13

**Authors:** George Efstathiou

**Affiliations:** ^1^ Kavli Institute for Cosmology, Madingley Road, Cambridge

**Keywords:** dark matter, dark energy, inflation

## Abstract

Observations of the cosmic microwave background (CMB) radiation are described with remarkable accuracy by the six-parameter ΛCDM cosmology. However, the key ingredients of this model, namely dark matter, dark energy and cosmic inflation are not understood at a fundamental level. It is, therefore, important to investigate tensions between the CMB and other cosmological probes. I will review aspects of tensions with direct measurements of the Hubble constant $H_{0}$, measurements of weak gravitational lensing, and the recent hints of evolving dark energy reported by the Dark Energy Spectroscopic Instrument (DESI) collaboration.

This article is part of the discussion meeting issue ‘Challenging the standard cosmological model’.

## 1. Introduction

The field of cosmology has been fortunate to have a major satellite mission dedicated to measuring the cosmic microwave background (CMB) in each of the last three decades. NASA’s Cosmic Background Explorer (COBE) led to the discovery of CMB anisotropies in 1992 [1]. NASA’s Wilkinson Microwave Anisotropy Probe, released its first results in 2003 [2] and established the six-parameter ΛCDM cosmology in the form that we know it today. The European Space Agency (ESA) *Planck* satellite was launched in 2009 and the final results from the *Planck* collaboration were presented in 2018 [3]. An overview paper summarizing the cosmological legacy of the *Planck* mission [4] concluded: *The six-parameter ΛCDM model continues to provide an excellent fit to the cosmic microwave background data at high and low redshift, describing the cosmological information in over a billion map pixels with just six parameters*.

In this contribution to the Royal Society Discussion Meeting, I will review whether the above quotation is still justified today. I will assume throughout that the Universe is homogeneous and isotropic on large scales, since this is a key ingredient of the ΛCDM cosmology and is supported by observations of the CMB. It is, of course, important to test the assumptions of homogeneity and isotropy of our Universe using different types of data. Such tests are described by others at this meeting.

## 2. The exquisite fit of to ΛCDM CMB anisotropies

**Figure 1.**
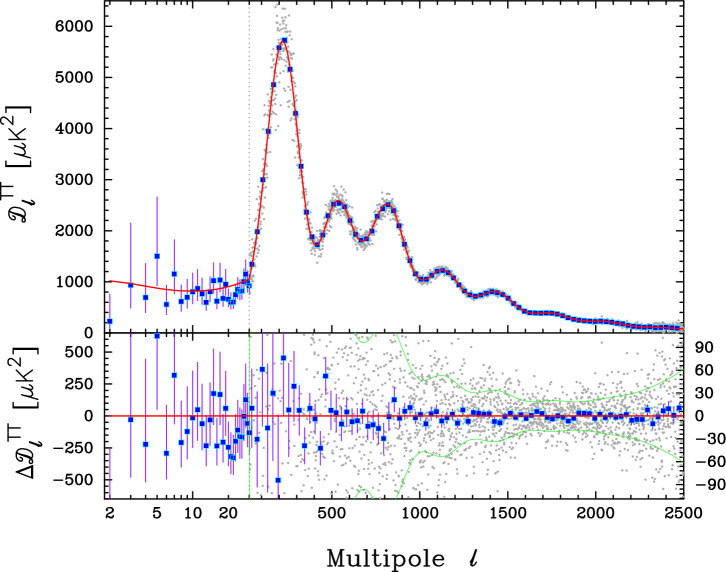
The upper panel shows the *Planck* CMB temperature power spectrum and the lower panel shows the residuals with respect to the power spectrum of the base six-parameter ΛCDM model fitted to the TTTEEE spectra (shown by the red line in the upper panel). The multipole scale is logarithmic over the multipole range 2–29 and linear at higher multipoles. The power spectrum computed over 86% of the sky from the Commander component separated maps [10] is shown over the multipole range 2–29 together with asymmetrical 68% error bars. The foreground corrected frequency average power spectrum computed from the NPIPE [9] *Planck* maps, averaged in multipole bins of width Δℓ=30, are plotted as the blue points. The faint grey points show the power spectrum multipole-by-multipole. The error bars show 1σ errors on the band powers computed from the diagonals of the high multipole covariance matrix. The green lines plotted in the lower panel show the ±1σ error ranges for the grey points.

**Figure 2.**
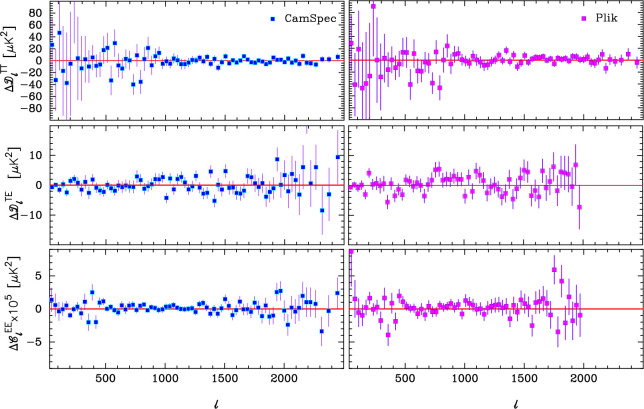
Residuals of the TT, TE and EE spectra relative to the best fit ΛCDM model plotted in figure 1. Residuals for NPIPE *CamSpec* spectra are shown in the left-hand panels. Residuals for the *Plik* spectra, as used in the baseline 2018 *Planck* TTTEEE likelihood, relative to the same cosmology are shown in the right-hand panels. (Adapted from [8]).

Following the 2018 *Planck* data release, in collaboration with Steven Gratton and Erik Rosenberg I began a programme to extract more information from the *Planck* power spectra by modifying the *CamSpec* pipeline [5] to use larger sky areas and dust-cleaned spectra [6,7]. The foreground corrected, frequency averaged, temperature power spectrum from the most recent iteration [8] based on the NPIPE *Planck* maps [9] is shown in figure 1. The high multipole power spectrum shown in this plot is effectively a full mission average of the 143×143, 143×217 and 217×217 power spectra computed over 80% of the sky. The cosmological parameters of the base six-parameter ΛCDM cosmology computed from this analysis are almost identical to those reported in [3]. However, the residuals with respect to the best-fit model are substantially smaller. This is true for each of the TT, TE and EE spectra as illustrated in figure 2.

This is a significant result. In our attempts to extract more information from *Planck*, the temperature and polarization spectra lock on even more accurately to the base ΛCDM cosmology. There is no evidence for any new physics beyond ΛCDM. Indications of anomalies, such as the excess smoothing of the TT acoustic peaks (quantified by the phenomenological parameter $A_{L}$), variations in cosmological parameters as a function of multipole [11] and features in the *Planck* spectra, e.g. [12], all decrease in statistical significance, consistent with the behaviour expected of statistical fluctuations. This conclusion is strengthened further by the extremely good agreement between the *Planck* ΛCDM best-fit model and the TE and EE spectra extending to high multipoles measured by the Atacama Cosmology Telescope (ACT) and by the South Pole Telescope (SPT) [13,14].

If it is argued that new physics beyond ΛCDM is required to explain, for example, distance scale measurements of the Hubble constant (the ’Hubble tension’), galaxy weak lensing measurements, (the ’$S_{8}$ tension’), evolving dark energy, or bulk flow anomalies (as described elsewhere in this volume) then that new physics must reproduce the CMB anisotropies of the base ΛCDM cosmology to extraordinarily high precision. Additional data beyond CMB measurements, therefore, become critical in assessing such challenges to ΛCDM .

## 3. The Hubble tension

Fitting the base ΛCDM model to the TTTEEE spectra of figures 1 and 2 combined with the Commander TT and SIMALL EE likelihoods at ℓ<30 [10] (I will use the terminology *Planck* TTTEEE to refer to this combination of the high and low multipole likelihoods), we find a Hubble constant of


(3.1) $$H_{0}=67.43\pm 0.49\;km\;s^{-1}{Mpc}^{-1}.$$

In contrast, the latest value of the Hubble constant measured by the SH0ES collaboration based on Cepheid variables and Type Ia supernovae (SN) is


(3.2) $$H_{0}=73.01\pm 0.99\;km\;s^{-1}{Mpc}^{-1},$$

[15]. These two numbers differ by more than 5σ, a discrepancy that has become known as the ’Hubble tension’—for recent reviews see [16–19].

It is extremely unlikely that the *Planck* value of 3.1 is wrong. There is a huge degree of redundancy in the *Planck* data and so there are many different ways in which the data can be partitioned. For example, the TE spectra alone (which are free of extragalactic foregrounds) give an $H_{0}$ consistent with equation (3.1) and with comparable accuracy. Furthermore, the high-resolution ground-based experiments give independent estimates of the Hubble parameter for the ΛCDM cosmology that are consistent with the *Planck* value and differ from the SH0ES value by many standard deviations ($H_{0}=67.6\pm 1.1\;km\;s^{-1}Mpc^{-1}$ for ACT TTTEEE combined with WMAP [20]; $H_{0}=68.3\pm 1.5\;km\;s^{-1}Mpc^{-1}$ for SPT = 3G TTTEEE, [21]). It is, therefore, reasonable to conclude that either the ΛCDM model is missing some new physics or the SH0ES estimate is biased in some way. Freedman, in this volume, presents new JWST observations of Cepheids, tip of the red giant branch and carbon-rich asymptotic giant branch stars to infer $H_{0}=69.8\pm 1.9\;km\;s^{-1}Mpc^{-1}$, slightly lower than the SH0ES value and consistent with the CMB value. On the other hand, JWST Cepheid photometry by the SH0ES team is in very good agreement with their earlier HST results, effectively eliminating systematics associated with crowded field photometry as the source of the tension [22,23]. Evidently, more work needs to be done to achieve a consensus between Freedman and collaborators and the SH0ES team. For the rest of this section I will take the SH0ES result at face value and discuss whether it is possible to modify ΛCDM to explain their value of $H_{0}$.

To begin, table 1 lists the posteriors for $H_{0}$ for variants of ΛCDM from the grid of models discussed in [3]; $m_{\nu }$ is the mass of a single massive neutrino eigenstate (fixed to 0.06eV in the base model, as expected for a normal hierarchy), $N_{\nu }$ is the number of neutrino/neutrino-like relativistic species (fixed to 3.046 in the base model), $m_{\mathrm{str}}$ adds a massive sterile neutrino, $n_{\mathrm{run}}$ adds a running of the scalar spectral index, $\Omega_{k}$ is the spatial curvature and the parameters $w_{0}$ and $w_{a}$ model dynamical dark energy (see §5). In the latter two variants, the CMB anisotropies suffer a large geometrical degeneracy [24] and cannot be determined $H_{0}$ accurately. Thus, the third column adds baryon acoustic oscillation (BAO) measurements as described in [3] to break the geometrical degeneracy. *There is not even a hint of movement towards the SH0ES value of $H_{0}$ in any of these variants*.

**Table 1.** Values of the Hubble constant with 1σ errors for extensions to ΛCDM.

model	*Planck* TTTEEE	*Planck* TTTEEE+BAO
ΛCDM	67.44±0.58	67.69±0.42
ΛCDM+ $m_{\nu }$	66.8±1.2	67.8±0.6
ΛCDM+ $N_{\nu }$	66.4±1.6	67.4±1.2
ΛCDM+ $m_{\nu }+N_{\nu }$	$66.1_{-1.6}^{+1.9}$	67.5±1.2
ΛCDM+ $m_{str}+N_{\nu }$	67.1±0.7	$67.89_{-0.69}^{+0.45}$
ΛCDM + $n_{run}$	67.25±0.6	67.66±0.45
ΛCDM+ $\Omega_{k}$	56±4	67.9±0.7
ΛCDM+ $w_{0}+w_{a}$	—	64.9±2.1

As discussed in §2, the *Planck* power spectra are extremely well fitted by the base ΛCDM cosmology. The $H_{0}$ posteriors of these variants peak at values close to that of base ΛCDM, but additional model complexity can introduce degeneracies that increase the error in $H_{0}$. Almost all proposed cosmological 'solutions’ to the $H_{0}$ tension (see [25] for a review) are of this type, i.e. favouring an $H_{0}$ that is close to the value of base ΛCDM but increasing the error because of internal parameter degeneracies. Furthermore, because the CMB is so well fitted by the base ΛCDM, the interpretation of theoretical solutions to the $H_{0}$ tension becomes sensitive to the use, and sometimes misuse, of supplementary astrophysical data (see e.g. [26]).

It is well known that by combining BAO measurements, the magnitude-redshift relation of type Ia SN, and the CMB value of the sound horizon $r_{d}$, it is possible to construct an inverse distance ladder for $H_{0}$ see e.g. [27–29]. In fact, it is not even necessary to assume ΛCDM since BAO and Type Ia SN strongly constrain the background expansion history to be close to that of ΛCDM irrespective of dynamics [27,30,31]. Modifications to ΛCDM at low redshift, for example, adding interactions between dark matter and dark energy, dynamical dark energy or decaying dark matter cannot significantly affect the inverse distance ladder. For example [31], assume the *Planck* value $r_{d}=147.27\pm 0.31\mathrm{Mpc}$ and use BAO measurements together with the Pantheon SN sample [32] to infer, in a model independent way:


(3.3) $$H_{0}=68.42\pm 0.88\;km\;s^{-1}{Mpc}^{-1},\;inverse\;distance\;ladder,\;\;Planck\;r_{d}\;,$$

which is in tension with SH0ES at approximately the 3.5σ level. This suggests that the Hubble tension requires a mechanism to lower the sound horizon (see e.g. [33]). However, the ΛCDM model is remarkably consistent. For example, [34] bypass the CMB estimate of $r_{d}$ using BAO measurements and constraints from big bang nucleosynthesis to infer $H_{0}=67.42_{-0.94}^{+0.86}\;km\;s^{-1}Mpc^{-1}$, while [35] uses full-shape galaxy power spectrum (sensitive to the scale of matter-radiation equality) with *Planck* CMB lensing and Type Ia SN measurements to infer $H_{0}=64.8_{-2.5}^{+2.2}\;km\;s^{-1}Mpc^{-1}$, again without assuming the *Planck* value for $r_{d}$.

Early dark energy (EDE) is an attempt to preserve the physics of ΛCDM at both high and low redshift, by introducing a ’confusiton’—a scalar field ϕ that is dynamically important only at around the time of recombination (see the reviews by [36,37], and references therein). Here, I will review some results from [8] in which we considered a scalar field evolving in an axion-like potential


(3.4) $$\begin{array}{ccc} & V(\theta )=m^{2}f^{2}[1-\cos(\theta ){]}^{3}, & \end{array}$$

where m represents the axion mass, f the axion decay constant, and θ≡ϕ/f is a re-normalized field variable defined such that −π≤θ≤π. This potential has been considered by many authors e.g. [38,39]. and provides a flexible model with which to illustrate the observational consequences of EDE. This model adds three parameters to base ΛCDM which (following [39]) we choose to be the critical redshift $z_{c}$ at which the scalar field starts to roll, the fractional contribution of EDE to the total energy density at that redshift $f_{\mathrm{EDE}}(z_{c})$ and the initial field value $\theta_{i}$.

**Figure 3.**
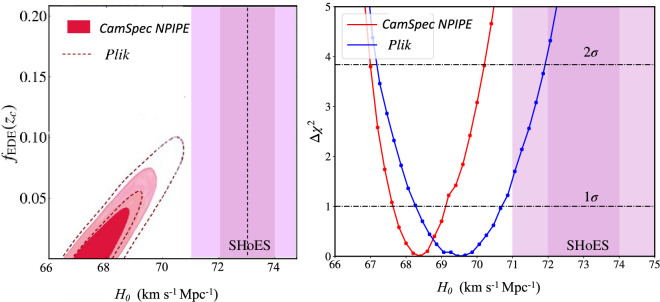
Left-hand plot shows Bayesian constraints on $H_{0}$ and the EDE parameter $f_{\mathrm{EDE}}(z_{c})$ using two *Planck* likelihoods in combination with BAO and SN data as described in the text. The right-hand plot shows profile likelihoods of $H_{0}$. The purple-shaded regions show the 1 and 2σ ranges of the SHoES measurement of $H_{0}$. (Adapted from [8]).

The left-hand panel of figure 3 shows Bayesian constraints on the parameters $f_{EDE}(z_{c})$ and $H_{0}$ fitted to the *Planck* data combined with BAO and SN data from the updated Pantheon+ SN catalogue [40]. For details of the data used see [8]. The red contours show constraints obtained using the *CamSpec* NPIPE likelihood at multipoles ≥30 (with power spectrum residuals shown in figure 2) and the dotted contours show the constraints obtained using the 2018 *Plik* likelihood in place of *CamSpec*. The right-hand panel shows profile likelihoods of $H_{0}$ which are independent of priors (see e.g. [41]). Both *Planck* likelihoods disfavour EDE and are in tension with the SH0ES value of $H_{0}$. The key conclusion to be drawn from figure 3 is that an improvement in the *Planck* likelihood leads to even greater tension with the SH0ES value of $H_{0}$ and favours parameters close to those of base ΛCDM (irrespective of statistical methodology and choice of priors). EDE, as a solution to the Hubble tension, is quite strongly disfavoured by the data. Similar conclusions have been reached by [42] and [43] using different data combinations.

In summary, observational data probing both high and low redshifts set such strong constraints that it is difficult to construct a plausible theory^1^ to match the SH0ES value of $H_{0}$ see also [45]. Any such theory must involve parameter degeneracies that combine fortuitously to mimic the six-parameter ΛCDM cosmology to high accuracy. This is why I regard the Hubble tension as such a frustrating challenge to ΛCDM .

## 4. The $S_{8}$ tension

Surveys of weak galaxy lensing provide measures of the parameter combination^2^ $S_{8}=\sigma_{8}(\Omega_{m}/0.3{)}^{0.5}$, consistently finding values that are lower than that expected according to the *Planck* best fit ΛCDM cosmology. This discrepancy has become known as the ’$S_{8}$ tension’.

In addition to weak lensing, there are other ways of measuring the amplitude of the fluctuation spectrum. The perception has arisen that the $S_{8}$ tension reflects a difference between early time measures of the fluctuation spectrum such as the CMB and late time probes see e.g. [46]. Figure 4 from [50] challenges this perception. The plot shows constraints in the $S_{8}-\Omega_{m}$ plane from the Kilo-Degree Survey (KiDS) and Dark Energy Survey (DES) cosmic shear surveys. These contours sit low compared to the constraints from *Planck* (grey contours). The green contours show the constraints from *Planck* lensing combined with BAO and the CMB acoustic peak location parameter $\theta_{\mathrm{MC}}$. CMB lensing is caused by matter along the line of sight with a median redshift of z∼2, yet $S_{8}$ is consistent with the value inferred from the primary anisotropies. There is no evidence of a departure from the ΛCDM fluctuation growth rate between z∼1000 and z∼2. Furthermore, there is no evidence for ’gravitational slip’; photons are responding to the same gravitational potential as the matter. The purple contours show constraints from redshift space distortions (RSD) using the same galaxy and quasar survey RSD measurements as those used in [3]. These surveys cover the redshift range 0.1–1.5, overlapping in redshift with the weak lensing surveys. Although the errors are quite large, RSD are consistent with the primary CMB results with no evidence for a slowing of the linear growth rate with redshift.

**Figure 4.**
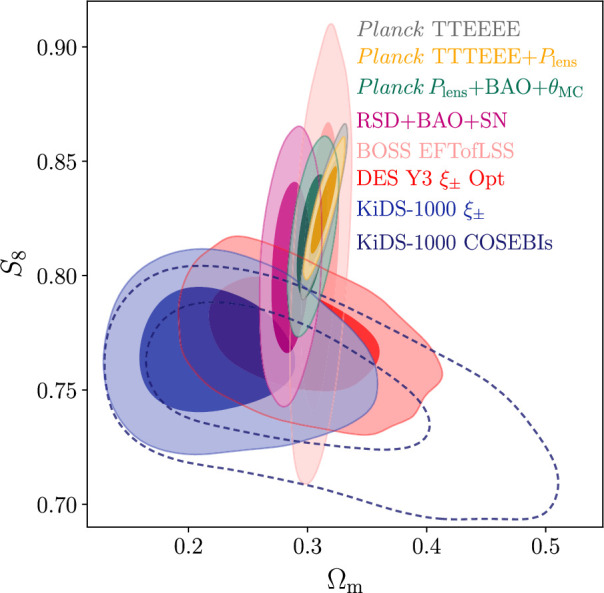
Contours showing 68 and 95% constraints in the $S_{8}-\Omega_{m}$ plane for various data assuming the six-parameter ΛCDM cosmology. The blue and navy (dashed) show the constraints from the KiDS $\xi_{\pm }$ and COSEBI statistics as analysed by [47], while the red shows that from the DES Y3 ΛCDM optimised $\xi_{\pm }$ analysis [48]. The yellow and grey contours show constraints from *Planck* TTTEEE with and without the addition of the *Planck* CMB lensing likelihood (Plens). The peach contours labelled EFTofLSS show constraints from the BOSS power spectrum and bispectrum effective field theory analysis of [49]. The magenta contours show constraints from RSD combined with BAO and SN measurements as described in [50]. The green contours show the constraint from the *Planck* lensing likelihood combined with BAO together with conservative priors on the acoustic peak location parameter $\theta_{\mathrm{MC}}$ (an approximation to the parameter $\theta_{*}$ defined in §5) and other cosmological parameters.

Several groups have developed ‘full-shape’ analyses based on effective field theory (EFT) descriptions of nonlinear perturbations e.g. [49,51–54]. The EFT analyses aim to constrain cosmological parameters independently of *Planck*. However, the nuisance parameters required to model perturbation theory, galaxy biasing and redshift space distortions, effectively down-weight information at wavenumbers $k≳0.2h^{-1}Mpc$. As a consequence of the restricted wavenumber range, the primordial spectral index $n_{s}$ is poorly constrained in comparison to *Planck*. At the approximately 1–2σ level, EFT RSD depend on the choices of priors, particularly the parameter $n_{s}$. The peach coloured contours in figure 4 show results from the EFT power spectrum and bispectrum analysis of the Baryon Oscillation Spectroscopic Survey (BOSS) galaxy sample of [49]. These authors apply a simulation based correction for prior volume effects in the EFT analysis that bias $\sigma_{8}$ low by approximately 1σ if left uncorrected. The errors in $S_{8}$ from this analysis are large but, significantly, there is no evidence of tension with the *Planck* ΛCDM cosmology. The role of priors in RSD analyses is discussed further by [55–57].

Figure 4 suggests an alternative interpretation of the $S_{8}$ tension [50]. The CMB and RSD measurements probe spatial scales that are in the linear regime. In contrast, weak lensing measurements are dominated by scales that are highly nonlinear. If physical processes suppress the amplitude of the nonlinear spectrum on small scales over and above the expectations of a universe composed of collisionless matter, then it may be possible to explain the $S_{8}$ tension while preserving the predictions of the base ΛCDM cosmology on large scales where the density field is linear. Baryonic feedback is an obvious mechanism that could produce such a suppression (see e.g. [58–60], and references therein). A more speculative proposal, which should not be discounted, is that a suppression is caused by new physics in the dark sector, for example, a contribution from light axions (see e.g. [61], and references therein).

Reference [50] investigated the effects of power spectrum suppression by introducing the phenomenological model


(4.1) $$\begin{array}{ccc} & P_{m}(k,z)=P_{m}^{L}(k,z)+A_{\mathrm{mod}}[P_{m}^{\mathrm{NL}}(k,z)-P_{m}^{L}(k,z)]\;. & \end{array}$$

Here, $P_{m}(k,z)$ is the matter power spectrum at wavenumber k and redshift z, the superscript L denotes the linear theory power spectrum and NL denotes the dark matter nonlinear power spectrum with no baryonic feedback (as computed, for example, by Euclid Emulator [62] or HMCode2020, [63]). The parameter $A_{\mathrm{mod}}$ is a constant that describes suppression of the spectrum on nonlinear scales if $A_{\mathrm{mod}}<1$. This model, and its relationship to models of baryonic feedback are described in greater detail in the talk by Amon at this meeting. As summarized by [64], the *Planck* ΛCDM cosmology gives acceptable fits to the KiDS and DES Y3 cosmic shear data for values of $A_{\mathrm{mod}}$ in the range ∼0.75–0.9 depending on whether scale cuts are applied to the two-point statistics. The question of whether such values of $A_{\mathrm{mod}}$ can result from baryonic feedback is controversial see e.g. [65–68] and so further work is required to assess whether the proposal of [50] is viable. However, *it would only take one convincing measurement of a low value of $S_{8}$ on linear scales to falsify the proposal*.

**Figure 5.**
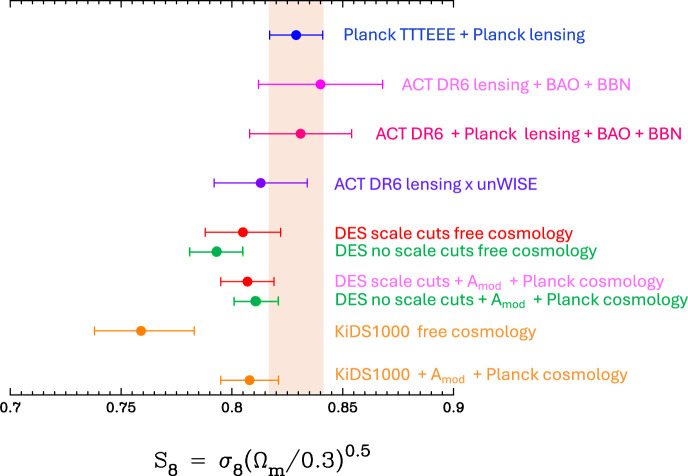
Summary of measurements of $S_{8}$ including new results from ACT DR6 CMB lensing measurements [69] and ACT DR6 lensing cross-correlate with unWISE galaxies [70]. The remaining entries show results for the DES and KiDS weak lensing surveys as described in the text.

Figure 5 includes two new measurements of $S_{8}$ on linear scales. The points labelled ACT DR6 show new results on CMB lensing from ACT Data Release 6 as described by [69]. These measurements are consistent with the *Planck* TTTEEE constraints on $S_{8}$ and with *Planck* lensing. The point labelled ACT DR6 lensing × unWISE shows the result of cross-correlating ACT DR6 lensing with galaxies from the unWISE catalogue spanning the redshift range z∼ 0.2–1.6 see [70], for details. Results from ACT DR6 are discussed in greater detail by Madhavacheril at this meeting (including preliminary results from cross-correlating ACT DR6 lensing with DESI luminous red galaxies (LRG).^3^

The remaining entries show results from [64] who re-analysed DES Y3 and KiDS−1000 $\xi_{\pm }$ cosmic shear measurements including the parameter $A_{\mathrm{mod}}$. The red points in figure 5 apply the ΛCDM optimized scale cuts to the DES Y3 $\xi_{\pm }$ measurements. As discussed in [50] the DES Y3 analysis applied angular scale cuts to reduce biases caused by baryonic feedback using the EAGLE [72] and OWLS-AGN [58] hydrodynamic simulations as a reference (see [73]). The green points labelled ’no scale cuts’ use all of the DES Y3 $\xi_{\pm }$ data points with angular separation $\geq 2.5^{\prime }$. KiDS−1000 [47] make more aggressive use of small scales, retaining all scales with $\theta \geq 0.5^{\prime }$ in $\xi_{+}$ and $\theta \geq 4^{\prime }$ in $\xi_{-}$. For each survey and choice of scale-cuts we show results for $S_{8}$ allowing cosmological parameters to vary with uninformative priors (labelled ’free cosmology’), with a *Planck* ΛCDM prior on cosmological parameters (labelled ’*Planck* cosmology’) and with and without including the power spectrum suppression parameter $A_{\mathrm{mod}}$. Applying scale cuts to DES Y3 we see that allowing $A_{\mathrm{mod}}$ (with best fit value $A_{\mathrm{mod}}=0.92\pm 0.10$) to vary has little effect. The weak lensing measurements on large angular scales are consistent with the *Planck* ΛCDM cosmology. However, if the small scale data are included, consistency of DES Y3 data with the *Planck* ΛCDM cosmology requires a suppression of the nonlinear power spectrum with $A_{\mathrm{mod}}=0.86\pm 0.05$. The KiDS−1000 data probe even smaller scales and require $A_{\mathrm{mod}}=0.75\pm 0.07$ to match the *Planck* cosmology. If the suppression is interpreted in terms of baryonic feedback, the latter two values of $A_{\mathrm{mod}}$ imply substantially stronger feedback than expected from recent hydrodynamical simulations e.g. [67,74].

Clearly an accurate model of the nonlinear matter power spectrum, including the amplitude and scale dependence of the effects of baryonic feedback, is required to infer an unbiased value of $S_{8}$ from cosmic shear surveys.^4^ In the future it will become possible to constrain baryonic feedback empirically using cross-correlations of weak lensing and measurements of the kinetic and thermal Sunyaev–Zeldovich effects e.g. [68,77,78]. Such studies will also provide valuable additional constraints on the modelling of feedback in numerical hydrodynamic simulations. RSD measurements from DESI should provide a decisive test on whether the fluctuation growth rate at late times is compatible with the *Planck* ΛCDM cosmology.

## 5. Evolving dark energy?

Just before this meeting, BAO measurements from the first year of DESI observations were submitted to the archive [79,80]. Combining these BAO measurements with CMB observations and various SN catalogues, the DESI team report evidence for a time varying equation of state [81], though they caution: *it is important to thoroughly examine unaccounted-for sources of systematic uncertainties or inconsistencies between the different datasets that might be contributing to these results*. The DESI results are discussed by Palanque–Delabrouille at this meeting. Here, I will make some general remarks on whether the DESI results pose a challenge for ΛCDM .

The DESI team parameterize the evolution of the dark energy equation-of-state (EoS) with redshift z as


(5.1) $$\begin{array}{ccc} & w(z)=w_{0}+w_{a}\left(\frac{z}{1+z}\right), & \end{array}$$

introducing two additional parameters, $w_{0}$ and $w_{a}$ to the base ΛCDM cosmology. This parameterization has the virtue of simplicity [82] but since the redshift dependence is constrained at both high and low redshift, one must be cautious about interpreting constraints in $w_{0}-w_{a}$ space in terms of phantom crossing points (i.e. transitions of the EoS to phantom-like behaviour with w(z)<−1). This is because observational data constrain the EoS over a limited redshift range. The behaviour of w(z) in the model of equation (5.1) outside that limited range is a consequence of the parameterization rather than the data, see e.g. [83–85]. We will therefore treat equation (5.1) as a purely phenomenological parameterization.

**Figure 6.**
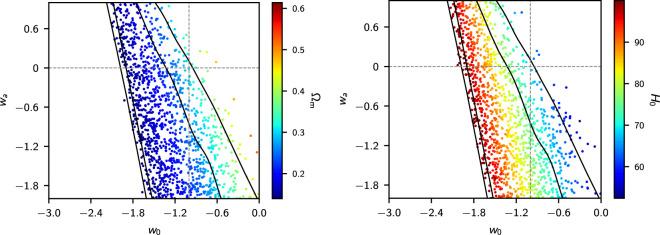
Contraints on $w_{0}-w_{a}$ from the NPIPE *Planck* TTTEEE likelihood colour coded by the value of $\Omega_{m}$ (left-hand plot) and $H_{0}$ (right-hand plot, with $H_{0}$ in units of $km\;s^{-1}Mpc^{-1}$). This plot shows that the CMB provides very weak constraints on $w_{0}-w_{a}$ because of a large geometrical degeneracy.

Figure 6 shows the constraints from *Planck* TTTEEE colour coded by $\Omega_{m}$ and $H_{0}$. The parameters $w_{0},w_{a}$ are highly degenerate, reflecting the geometrical degeneracy $\theta_{*}=r_{*}/D_{M}(z_{*})$, where $\theta_{*}$ is the acoustic peak location parameter, $r_{*}$ is the sound horizon at the time of recombination $z_{*}$ and $D_{M}(z_{*})$ is the comoving angular diameter distance to $z_{*}$. The lines in figure 6 show the geometrical degeneracy $r_{*}/D_{M}(z_{*})=constant$ for fixed values of $\Omega_{m}$ and $H_{0}$. To maintain the acoustic peak structure, $\omega_{b}=\Omega_{b}h^{2}$, $\omega_{c}=\Omega_{c}h^{2}$ (and hence $\omega_{m}=\omega_{b}+\omega_{c}$) must be approximately constant, so the spread in figure 6 is set approximately by the error in $\omega_{m}$ for the base ΛCDM cosmology. This is why high values of $H_{0}$ in the right-hand panel correspond to low values of $\Omega_{m}$ in the left-hand panel. For $w_{a}=0$, the MCMC samples are skewed to values $w_{0}<-1$. This is caused mainly by the slight TT power deficit at ℓ≲30 compared to the best fit base ΛCDM model (see figure 1), which tends to pull $w_{0}$ into the phantom domain via the integrated Sachs-Wolfe effect, though at low statistical significance [86]. Figure 6 shows why $H_{0}$ is unconstrained by *Planck* TTTEEE if $w_{0},w_{a}$ are added as parameters to ΛCDM (cf. table 1). Evidently CMB anisotropies alone cannot constrain $w_{0},w_{a}$. Any evidence for evolving dark energy must therefore come from supplementary data.

**Figure 7.**
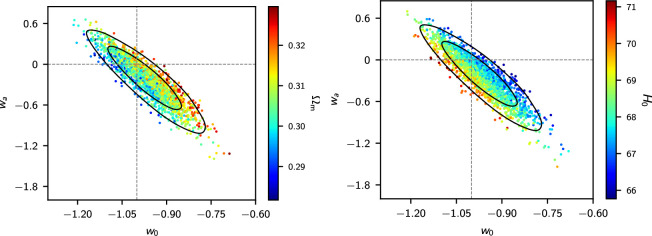
Constraints on $w_{0}-w_{a}$ for *Planck* TTTEEE combined with pre-DESI BAO measurements (as used in [3]) combined with the Pantheon SN catalogue. The MCMC samples are colour coded by the value of $\Omega_{m}$ (left-hand plot) and $H_{0}$ (right-hand plot, with $H_{0}$ in units of $km\;s^{-1}Mpc^{-1}$). The cosmological constant corresponds to the intersection of the two dotted lines.

Figure 7 adds BAO data, as described in [3] (dominated statistically by BOSS DR12) and the Pantheon SN sample. As noted in [3], there is no evidence for evolving dark energy from these data. The conclusions of the DESI team must therefore be a consequence of differences in the BAO and/or SN data.

**Figure 8.**
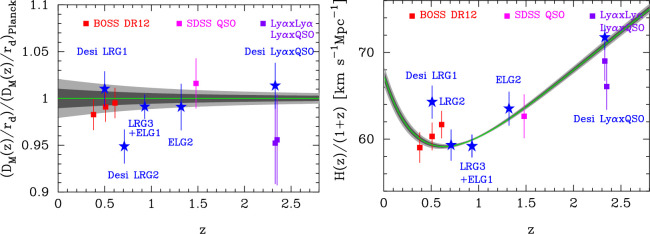
Comparison of BOSS/SDSS and DESI measurements of $D_{M}(z)$ and H(z). The green lines show the predictions of the best fit *Planck* base ΛCDM cosmology and the grey bands show 1 and 2σ errors. The BOSS/SDSS measurements are from the following sources: BOSS DR12 [87], SDSS QSO [88], BOSS Lyα×Lyα, Lyα×QSO [89,90]. The DESI measurements are from [79,80].

Figure 8 compares the DESI BAO results with the earlier BOSS/SDSS measurements with the predictions of the *Planck* base ΛCDM model. I plot only measurements which have high enough signal to noise to measure both H(z) and $D_{M}(z)$ from the BAO scale along and perpendicular to the line-of-sight. The BOSS/SDSS measurements are in good agreement with the *Planck* model. However, as noted by the DESI team, there are two outliers amongst the DESI measurements; these are $D_{M}$ for the DESI LRG2 sample (⟨z⟩=0.71), which sits low compared to the *Planck* prediction by approximately 2.6σ and H(z) for the DESI LRG1 sample (⟨z⟩=0.51), which sits high compared to the *Planck* prediction by approximately 2.8σ. As an approximate guide, if the errors are assumed to be Gaussian and uncorrelated, the probability of getting two such deviant points out of 22 points is approximately 1.8%. This is unusual, but not excessively so. Importantly, the two deviant DESI points are in tension with the results from BOSS RD12, which measure $D_{M}$ and H(z) more accurately than DESI at similar redshifts.^5^ Furthermore, the new data points do not reinforce any coherent pattern in the earler BAO measurements that might indicate a deviation from ΛCDM , suggesting that the DESI outliers are just statistical fluctuations.

The results presented in [81] strongly suggest that the evidence for evolving dark energy is driven by the new SN catalogues. As with the CMB, the DESI BAO measurements are strongly degenerate in the parameters $w_{0}-w_{a}$ and show no significant preference for evolving dark energy.^6^ It is only when the supernova samples are added to *Planck* CMB and DESI BAO that they see a pull towards evolving dark energy, finding a preference for evolving dark energy compared to ΛCDM (using $\Delta \chi_{\mathrm{MAP}}^{2}$ as a tension metric) at approximately the 2.5σ (Pantheon+), 3.5σ (Union 3, [91]) and 3.8σ level (DESY5 SN, [92]. In summary, it is important to scrutinize the SN samples (particularly the new Union 3 and DESY5 samples) to rule out the possibility that the DESI ‘dark energy tension’ is caused by systematic errors in the SN data.^7^

## 6. Conclusions

The six-parameter ΛCDM cosmology is remarkably successful. Yet we have little understanding at a fundamental level of the three key features of the model—inflation, dark matter and dark energy. It is therefore possible that the tensions discussed in this article are caused by new physics. I have emphasised the fact that the six-parameter ΛCDM cosmology agrees to high precision with observations of the CMB anisotropies and with CMB lensing. Observational evidence for departures from ΛCDM is therefore conditional on the fidelity of other types of astrophysical data. Fortunately, there are many new projects underway that should not only clarify the tensions discussed here but should provide stringent new tests of the ΛCDM cosmology.

## Data Availability

This article has no additional data.

## Footnotes

^1^
It is possible to construct contrived models to evade these problems. For example it has been suggested that a sudden transition in the value of the gravitational constant within the last 100 million years might have led to a dimming of nearby Type Ia SN e.g. [44].

^2^
Where $\Omega_{m}$ is the present day matter density in units of the critical density, $\sigma_{8}$ is the root-mean-square linear amplitude of the matter fluctuation spectrum in spheres of radius $8h^{-1}\mathrm{Mpc}$ extrapolated to the present day, and h is the value of the Hubble constant $H_{0}$ in units of $100\mathrm{km}s^{-1}{\mathrm{Mpc}}^{-1}$.

^3^
Which can be compared to the cross-correlation analysis of DESI LRG with *Planck* lensing which showed hints of a low value of $S_{8}$ [71].

^4^
Differences in the amplitude of the $S_{8}$ tension reported in the literature [47,66,75,76] are caused, in part, by differences in scale cuts and in the modelling of baryonic feedback.

^5^
In contrast, the DESI Lyα×QSO measurement improves significantly on the earlier BOSS/SDSS constraint, and comes into even better agreement with the *Planck* ΛCDM model.

^6^
The DESI team use $\Delta \chi_{\mathrm{MAP}}^{2}$ as a tension metric, which is the difference in $\chi^{2}$ for the maximum posterior allowing $w_{0}$ and $w_{a}$ to vary relative to $\chi^{2}$ for ΛCDM (i.e. $w_{0}=-1$, $w_{a}=0$). For DESI BAO, they find $\Delta \chi_{\mathrm{MAP}}^{2}=-3.7$.

^7^
After the first version of this paper was submitted I compared the photometry of SN common to both the Pantheon+ and the DESY5 catalogues. The results suggest that the pull towards evolving dark energy using DES5Y may be a consequence of a systematic error in matching the photometry of nearby SN with the more distant SN observed by DES [93].
